# First observation in a non-endemic country (Togo) of *Penicillium marneffei* infection in a human immunodeficiency virus-infected patient: a case report

**DOI:** 10.1186/1756-0500-6-506

**Published:** 2013-12-04

**Authors:** Akouda Akessiwe Patassi, Bayaki Saka, Dadja Essoya Landoh, Awerou Kotosso, Koudjo Mawu, Wemboo Afiwa Halatoko, Majesté Ihou Wateba, Komi Adjoh, Osseni Tidjani, Dominique Salmon, Palokinam Pitché

**Affiliations:** 1 Service des Maladies Infectieuses et de Pneumologie, CHU Sylvanus Olympio, Lomé, Togo; 2 Service de Dermatologie, CHU Sylvanus Olympio, Lomé, Togo; 3 Division de l’Epidémiologie, Ministère de la Santé, BP: 1396 Lomé, Togo; 4 Laboratoire de Mycologie et Parasitologie, CHU Sylvanus Olympio, Lomé, Togo; 5 Institut National d’Hygiène, Ministère de la Santé, Lomé, Togo; 6 Service des Maladies Infectieuses et Tropicales, Hôpital Cochin, AP-HP, Paris, France

**Keywords:** *Penicillium marneffei*, Human Immunodeficiency Virus (HIV), Togo

## Abstract

**Background:**

Infection with *Penicillium marneffei* is a common opportunistic infection in Southeast Asia where it is endemic. We report a case of *Penicillium marneffei* infection with fatal outcome in a Togolese woman infected with Human Immunodeficiency Virus (HIV).

**Case presentation:**

A 45-years-old patient, infected with Human Immunodeficiency Virus had consulted for ongoing febrile pneumonia since two weeks. Clinical examination revealed fever of 38.5°C, dyspnea, pulmonary syndrome condensation and papulo-nodular of “molluscum contagiosum” like lesions located on the face, arms, neck and trunk. Sputum smear was negative for tuberculosis. The chest radiograph showed reticulonodular opacities in the right upper and middle lobes and two caves in the right hilar region. The CD4 count was 6 cells/mm^3^ after a year of antiretroviral treatment (Zidovudine-Lamivudine-Efavirenz). She was treated as smear negative pulmonary tuberculosis after a lack of gentamicin and amoxicillin plus clavulanic acid response. Culture of skin samples and sputum had revealed the presence of *P. marneffei*. A treatment with ketoconazole 600 mg per day was initiated. After two weeks of treatment, there was a decrease in the size and number of papules and nodules, without any new lesions. We noted disappearance of cough and fever. The chest X-ray showed a decrease of pulmonary lesions. There was no reactivation of *P. marneffei* infection but the patient died from AIDS after two years of follow up.

**Conclusion:**

We report a case of *P. marneffei* infection in a HIV-infected patient in a non-endemic country. Clinicians should think of *P. marneffei* infection in all HIV-infected patients with “molluscum contagiosum” like lesions.

## Background

Infection with *P. marneffei* is an opportunistic infection occurring in immuno compromised patients [1]. It is endemic in South Asia and South-east Asia, where infection with *P. marneffei* ranks third behind tuberculosis and cryptococcosis in Thailand, and behind *Pneumocystis carinii* pneumonia (PCP) and pulmonary tuberculosis in Hong Kong among Human Immunodeficiency Virus (HIV) positive patients [2-4]. *P. marneffei* infection has also been reported in patients infected with HIV from non-endemic areas and who have stayed in endemic areas [5,6]. We report a case of infection with *P. marneffei* with fatal outcome in a Togolese woman infected with HIV at the clinical World Health Organization (WHO) stage III of acquired immunodeficiency syndrome (AIDS).

## Case presentation

**Figure 1:**
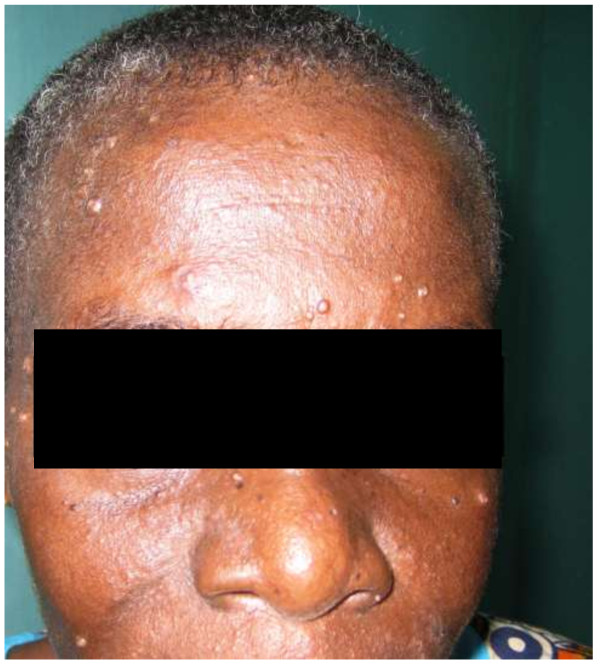
Papulo-nodular lesions “molluscum contagiosum” like on the face of the patient.

A 45-year-old woman had consulted on 7^th^ July 2008 in the Department of Infectious Diseases and Pneumology of the teaching hospital “Centre Hospitalier Universitaire” (CHU) Sylvanus Olympio in Lomé for ongoing fever, cough, dyspnea, since two weeks before admission. She was known to be HIV positive and was taking antiretroviral treatment (Zidovudine-Lamivudine-Efavirenz) associated with cotrimoxazole since July 2007. The CD4 count was 12 cells/mm^3^ at the beginning of antiretroviral treatment. She has always lived in Togo and has never traveled outside the country. The clinical examination of the patient revealed a poor general condition and nutritional status, a body temperature of 38.5°C, respiratory rate of 48 breaths/min, a pulse at 78 beats/minute and blood pressure at 110/70 mm Hg. We noted a thrush and a scar of the right intercostal zona. On the face of the patient, we noted translucent papular and nodular “molluscum contagiosum like” lesion with a small central depression (Figure 1).

There was also a syndrome of pulmonary consolidation characterized by increased vocal vibrations at the right lung, dullness at the base of right lung crackling rales at both lung bases.

Chest radiography showed reticulonodular opacities in upper and middle right lobes and two caves in the right hilar region. The CD4 count was 6 cells/mm^3^, the hemoglobin rate was 10 g/dl and the leukocytes count was 2 × 10^3^ cells/μl. Smear was negative.

The patient was treated as bacterial pneumonia by the combination of amoxicillin-clavulanic acid and gentamicin for ten days without success, then as smear negative pulmonary tuberculosis with two months combination of (rifampicin + isoniazid + pyrazinamide + ethambutol) followed by four months combination of (rifampicin + isoniazid) protocol.

**Figure 2:**
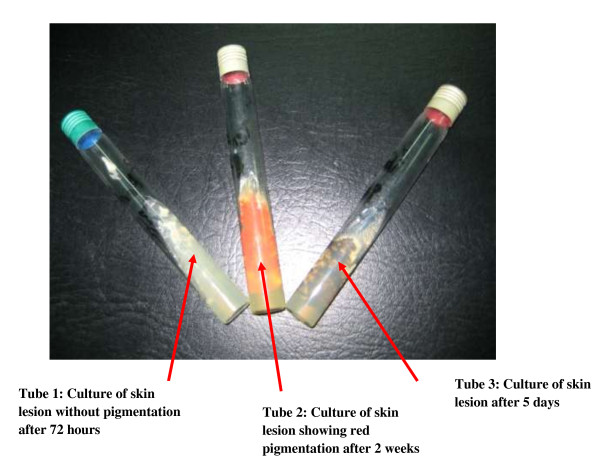
Red pigmentation after culture of skin lesion on Sabouraud medium without cycloheximide at 30° celsius.

**Figure 3:**
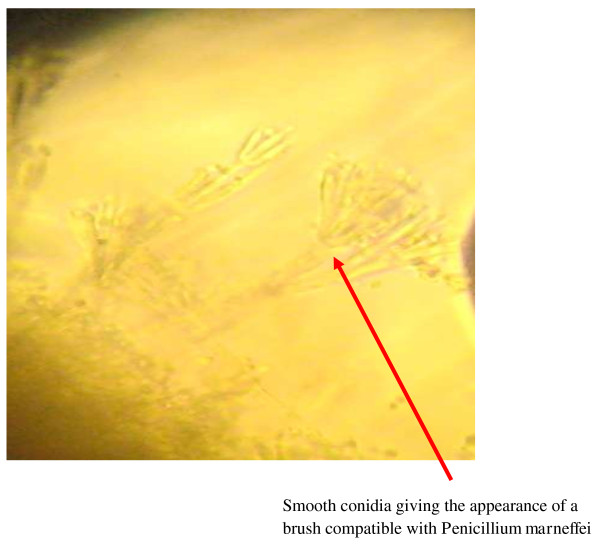
**Aspect of *Penicillium marneffei *** **at the microscopy observation.**

Fungal culture of skin scraping and sputum sample on Sabouraud medium with chloramphenicol without cycloheximide, revealed fluffy colonies with a red appearance grows after two weeks (Figure 2). Microscopic examination revealed hyaline filamentous forms with branches, sometimes with chains of smooth conidia giving the appearance of a brush compatible with *P. marneffei* (Figure 3).

A treatment with ketoconazole 600 mg per day was initiated. After two weeks of treatment, there was a decrease in the size and number of papules and nodules, without any new skin lesions. We noted disappearance of cough and fever. The chest X-ray showed a decrease of pulmonary lesions. There was no reactivation of *P. marneffei* infection but the patient died from AIDS after two years of follow up since his first consultation in the department of infectious diseases.

## Discussion

*Penicillium marneffei* infection has been reported as an endemic disease in patients with immunodeficiency in Thailand, China, Vietnam, Taiwan, Singapore and India.

The first reported case in West Africa occurred in a HIV positive patient originated from Ghana who had never traveled to tropical Asia [7]. Besides this case, several cases were reported among Africans who have traveled or resided in endemic countries [8-11]. In our opinion, the first case reported here in Togo and the case reported in Yo (Ghana) describe for the first time this infection in West Africa, an area that is far from the geographic reservoir. The natural reservoir, mode of transmission and the natural history of infection with *P. marneffei* are not clearly defined so far and remain research topics. The only known natural hosts are the bamboo rats (*Rhizomys* and *Cannomys spp.*) and humans [12]. *P. marneffei* can be isolated from soil and rarely from other environmental sources. Activities during the rainy season are also cited to be associated with the risk of exposure in endemic countries [13]. It is believed that humans are infected by inhaling infectious agent and rarely by direct animal contact. Inhaled spores are phagocytosed by pulmonary histiocytes that are disseminated in the body of the host and can cause a systemic infection. Person to person transmission of *P. marneffei* infection is not described nowadays.

The appearance of this infection in a Togolese patient remains enigmatic. Unfortunately, the epidemiological case investigation has not been made. But in 2000, the description of *P. marneffei* infection in a patient originating from Ghana which shares border with Togo may infer the existence of this fungal pathogen in these two countries. The development of maritime trade with the arrival of boats from Asia on the West African coasts could be ways of transmission and “colonization” of these coasts by the fungal.

The clinical features of penicilliosis are diverse and multifaceted and constitute a challenge in term of diagnostic for clinicians. The clinical outcome of penicilliosis can be fatal if it is not treated. Among the HIV-infected patients naive of antiretroviral therapy, penicilliosis is a sign of severe and late stage of HIV infection [12,14]. Our patient has immuno-clinical antiretroviral therapy failure (CD4 count 6 cells/mm^3^) corresponding to severe immunodeficiency. The patient has presented non-specific symptoms such as fever, anemia, productive cough and deterioration in general condition. These symptoms may reveal bacterial pneumonia or lung’s tuberculosis. These non-specific clinical signs and symptoms have justified the treatment with beta-lactam antibiotic associated with gentamicin and antituberculosis drugs. The evolution under this treatment was not favorable after two months. Moreover, the pulmonary manifestations of bacterial pneumonia do not associate skin lesion like molluscum contagiosum as found in the patient. Cutaneous manifestations features described in penicilliosis are translucent papular lesions, umbilicated with a central necrotic depression [13-16], similar to those presented by our patient on the face and trunk. These cutaneous manifestations are similar to skin lesions of cryptococcosis, histoplasmosis or molluscum contagiosum. However, the occurrence of these skin lesions in the context of fever, cough, dyspnea, weight lost and fatigue made us suggest infection with *P. marneffei* although the patient had always lived in Togo on the basis of a lack of clinical response to antibiotic treatment and culture on Sabouraud’s medium revealing a red pigment (Figure 2).

According to studies, culture from bone marrow, blood and skin samples are positive respectively at 100%, 76% and 90% of cases [13,17-20]. The identification of *P. marneffei* is based on the characteristics of its colony and microscopic morphology [16], with filamentous branches, sometimes with smooth conidia (Figure 3). The first description of this disease was made in a patient with Hodgkin’s lymphoma in Southeast Asia [7]. Before the first description of the disease in a HIV-infected patient in 1988 [8], penicilliosis was uncommon, with less than 40 cases reported [9,10].

*Penicillium marneffei* is highly sensitive to itraconazole, ketoconazole, voriconazole, miconazole, terbinafine and 5-fluorocytosine, amphotericin B, and less sensitive to fluconazole. The recommended treatment in penicilliosis is amphotericin B through intravenous route at a dose of 0.6 mg/kg per day for two weeks followed by 10 weeks of secondary prophylaxis with itraconazole (400 mg/day) [13,18]. Ketoconazole orally at a dose of 600 mg a day was the available treatment to our patient in all phases. This treatment was combined with highly active antiretroviral therapy. (Tenofovir/Lamivudine and boosted by Lopinavir).

This second case in West Africa raises the problem of fungus’ reservoir and the route of its transmission.

## Conclusion

Our observation reports a case of *P. marneffei* infection with fatal outcome in a patient infected with HIV in a non-endemic country. It invites clinicians to think of an infection with *P. marneffei* in all HIV-infected patients with lesions of molluscum contagiosum because only early diagnosis and proper treatment leads to a reduction in mortality associated with this disease.

## Consent

Written informed consent was obtained from the patient for publication of this case report and accompanying images. A copy of the written consent is available for review by the Editor-in-Chief of this journal.

## Competing interest

The authors declare that they have no competing interests.

## Authors’ contributions

AAP: is the primary health provider for the patient, conceived, designed, compiled the data for the article and wrote the article. BS and PP contributed to the clinical and therapeutic management from a dermatological point-of-view, they have participated in the interpretation of results, in writing and reviewing the manuscript and provided comments on the manuscript; AK, OT, MIW, KA: are also treating physician for the patient, they contributed in the manuscript design and editing. DEL: participated in the scientific management, interpretation, and the preparation of the final manuscript. KM and WAH: participated in laboratory confirmation and in reviewing the manuscript. DS: participated in interpretation of results, in writing and reviewing the manuscript and provided comments on the manuscript. All the authors have read and approved the final manuscript to be submitted for publication.
